# Polymorphism of serotonin transporter gene in male subjects with antisocial behavior and MMA fighters

**DOI:** 10.1038/s41398-018-0298-0

**Published:** 2018-11-15

**Authors:** Elena V. Cherepkova, Vladimir V. Maksimov, Lyubomir I. Aftanas

**Affiliations:** 1 Federal State Scientific Budgetary Institution “Scientific Research Institute of Physiology & Basic Medicine”, Novosibirsk, Russia; 2 Federal State Scientific Budgetary I Institution of Internal and Preventive Medicine, Novosibirsk, Russia; 3 Novosibirsk State University, Novosibirsk, Russia

## Abstract

In our study, the frequencies of serotonin transporter gene (5-HTT) polymorphisms and their combinations are compared in the healthy male subjects with antisocial behavior, in general, and in those with its particular forms, as well as in the reference group of MMA fighters. Subjects convicted of unlawful actions were classified into those convicted of violent crimes or non-violent ones. The group of subjects convicted of violent crimes was further subdivided into those convicted of murder, or robbery, or of inflicting grave body injuries. The group of MMA fighters was selected from the subjects without a prior history of antisocial behavior or criminal record in the subjects or their relatives. The frequency of D allele in the groups of convicted subjects and MMA fighters was higher, than in the population sample. Furthermore, the frequencies of D/D and 12/12 genotype combinations were shown to be higher in the group of convicted subjects, especially, in habitual criminals and those convicted of grave crimes or murder. The predisposition of MMA fighters to violent behavior and physical aggressive suppression of an opponent is successfully implemented in their professional career; however, this behavioral pattern appears to represent the controlled aggression.

## Introduction

The studies of twins behavior across the life span starting from their early childhood have demonstrated that the genetic factor has a considerable or major influence on the risk of developing antisocial behavior^1–3^. The genes of brain neurotransmitter systems including the serotonin system are believed to be the candidate genetic determinants of antisocial behavior^4–6^.

Serotonin transporter (5-hydroxytryptamine transporter; 5-НТТ; SLC6A4) is responsible for the re-uptake and transport of serotonin in presynaptic cleft, and it plays an important role in serotonergic regulation. The gene coding for 5-HTT is located on the long arm of chromosome 17 (17q11.1-q12).

Serotonin is a neurotransmitter involved in the regulation of complex behavioral patterns^7^. For example, the CSF concentration of serotonin metabolite is inversely correlated with aggressive and impulsive behavior^8^ or with difficulties in adjustment to military life^9–11^. In the studies of predisposition to neurological or psychiatric disorders, the polymorphism of 5-HTT gene also draws special attention^12–14^.

The genetic polymorphism of 5-HTT is associated with a 44-bp insertion or deletion localized in 5′-promoter region of SLC6A4 gene coding for serotonin transporter (5-HTTLPR, serotonin-transporter-linked polymorphic region). The polymorphic alleles are associated with the differential expression of the gene, with long (L) 5-HTTLPR allele being responsible for the higher transcription levels, as compared with the short one (S allele). The decreased expression of 5-HTT S (or SL) alleles is associated with an increased risk of development of antisocial behavior combined with aggression, impulsivity, and psychoactive substance abuse^15–18^.

High frequencies of short S allele and S/S genotype carriers were observed among the patients characterized by recurrent and overt physical violent behavior and the inclination to negative emotionality, including hostility^19^. In the study by Liao et al.^20^, the authors suggest that the low-activity S allele is linked to the excessively hostile criminal behavior in the male subjects of Chinese origin, and 5-HTT may underlie the mechanisms of hostile behavior.

Serotonin Transporter Intronic VNTR Enhancer (Stin2) located in intron 2 of 5-HTT gene contains 9, 10, or 12 repeats, each 16–17 bp long. Evidence is present that the allele with 12 tandem repeats is more functional, as far as the serotonergic transmission is concerned, compared with the 10-repeat allele^21^. The drug abusers, who are the carriers of a 10-repeat allele of 5-HTT gene and/or the 9-repeat VNTR polymorphism in DAT gene, are at extremely high risk of developing the antisocial personality disorder^22^.

On the other hand, the 10-repeat VNTR allele of 5-HTT gene was shown to be underrepresented among the Canadian children with extremal, stable, and pervasive aggression^23^. In our research, we investigate the contribution of 5-HTT VNTR polymorphism to the predisposition to antisocial behavior.

In the Russian population, the frequency of 5-HTT S/S genotype among the top class combat sportsmen (international class athletes and merited athletes) practicing Judo, Sambo, and Greco-Roman wrestling is higher, than in the controls^24^.

In particular, we make a comparative study of ID genotype and 5-HTT VNTR polymorphism frequencies in the healthy male subjects of Russian and Chechen origin, the subjects with particular forms of antisocial behavior (convicted of unlawful actions), and the comparison group of male mixed martial art (MMA) fighters.

## Methods

The study included four groups of male subjects, namely, two main study groups (one comprised of subjects convicted of unlawful actions and the other, which was intended to be a comparison group to the first one and which included the male MMA fighters) and two control groups (the general population sample and the cohort of male adolescent subjects).

The first group included 214 male subjects (age 19–64, mean age 38.2 ± 9.1), who committed the crimes of different gravity. All study participants were of Caucasian origin, 154 subjects (72%) identified themselves as the Russians, and 60 subjects (28%) as Chechens, in the survey questionnaire. About 2/3 of cases (63.3%) represented the repeat offenders.

Among the study participants, 166 subjects (77.6%) were convicted of the crimes of violence, including 88 subjects (53.3%) convicted of murder and 78 subjects (46.7%) convicted of robbery or inflicting serious physical injuries. The rest of 48 subjects (22.4%) were guilty of nonviolent crimes. All study participants were the inmates of Russian prisons.

Exclusion criteria included the symptoms of endogenous psychiatric disorders, serious organic CNS lesions, mental retardation, attention deficit hyperactivity disorder, serious somatic diseases, or reaction to heavy stress. An important inclusion criterion was the subject’s upbringing in a socially well-adjusted family in childhood and adolescence. Psychiatric surveys were performed by qualified psychiatrists.

The second study cohort, which was selected as a comparison group to the subjects with criminal behavior, included 107 athletes practicing MMA and demonstrating achievements in this combat sports, age 33 ± 6.1 and, mainly, of Russian origin. Of them, 70 athletes (65.4%) were demonstrating especially hard fighting style and were often ending the rounds/fights with knockdown/knockout, while usually inflicting serious body injuries on the opponent.

The subjects of this group lacked any psychiatric disorders or serious somatic diseases. Additionally, a key inclusion criterion was the lack of any unlawful actions in their background, as well, as the lack of criminal record in their relatives of both lineages (as certified by the law enforcement agencies). This reference/comparison cohort was included in the study, since both MMA fighters and the subjects with criminal behavior have a characteristic inclination toward risk behavior, which is thought to be the reason for them to choose the activities/occupation associated with high health and life risks.

The first control group included 528 male subjects of the Russian origin from Novosibirsk, Russia (mean age 56 ± 7.1), who were participated in the World Health Organization MONICA (multinational monitoring of trends and determinants in cardiovascular disease) project. The exclusion criterion was the presence of evident psychiatric disorder symptoms. The question about criminal record/conviction was not included in the questionnaire for this group. This group was randomly selected from the municipal voters list.

Of note, the subjects included in this general population sample group were not surveyed for their prior history of psychiatric/drug addiction disorders and their hereditary predisposition to such disorders, thus, the group may be heterogenous, as far as these factors are concerned. The contribution of genetic predisposition and the environmental factors in the development of criminal behavior is the matter of scientific debate with polarized opinions existing.

The second control group included 284 college and/or high school students (age 14–18, mean age 15.6 ± 0.9). All young subjects of the study were volunteer study participants, and for each adolescent study participant the informed consent form was completed by their parents.

Compliance with ethical standards. This research was carried out in accordance with The Russian Federal Law on personal data (No.152-FZ) of 27.07.2006, as well as with the International Ethical Guidelines, Declaration of Helsinki. Study was approved by the Bioethics Committee of the Federal State Scientific Budgetary Institution “Scientific Research Institute of Physiology & Basic Medicine” and by the Bioethic Committee of the Institute of Therapy and Prophylactic Medicine (Russian Academy of Medical Sciences, Siberian Branch).

The genetic study included the determination of frequencies of ID and VNTR polymorphic alleles of serotonin transporter gene (5-HTT) and the corresponding genotypes, following the protocol published for 5-HTT polymorphisms^25^.

Statistical analysis was performed using SPSS 11.5 software package. Initially, the frequencies of genotypes and alleles of the studied polymorphisms were determined in the control group in order to assess their agreement with the Hardy–Weinberg equilibrium (chi-square test). Then, the frequencies of genotypes and alleles of these polymorphisms were assessed in the groups of subjects with antisocial behavior and the MMA fighters (reference group). The differences in allelic and genotypic frequencies among the groups were evaluated with the help of contingency tables using Pearson’s chi-square test. When using the fourfold tables for the comparison of allelic and genotypic frequencies in the groups, we applied the two-sided exact Fisher’s test with Yates’ continuity correction. The relative risk for the carriers of a particular allele or genotype to fall into a certain group was calculated as the odds ratio.

## Results

The fact that both study cohorts were highly selected deserves special attention. MMA fighters are not as many, as the athletes practicing other sports, and we have been selecting the most effective fighters. The next level of selection was ethnic-based (only the athletes of Russian or Chechen origin were chosen), followed by another level of strict selection, when any subject with criminal record or the propensity to deviant behavior in the athletes themselves or in their close relatives was disqualified. Then, a psychiatric survey was performed, which disqualified some non-eligible individuals, as well. All participants of this group were brought up in socially well-adjusted families, and all of them were well educated. The group of convicts was also highly selected, and the individuals with the suspected psychiatric disorders have been rejected. In the group of convicts, all the participants were also brought up in well-adjusted or even well-doing families, all of them had high school education, and some of them also had their university education started or completed.

### ID polymorphism of serotonin transporter gene

**Fig. 1:**
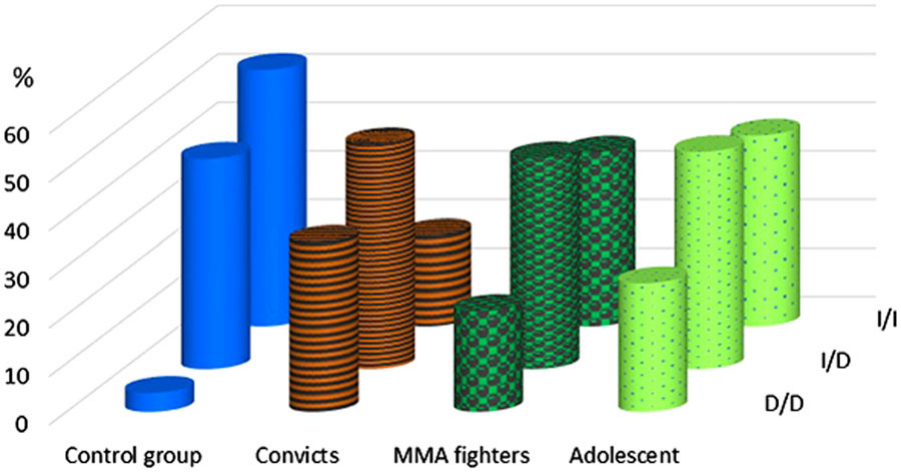
Genotype frequencies of ID polymorphism of HTT gene in the male subjects with antisocial behavior, the MMA fighters, the general population, and in adolescents. Study groups and combinations of ID alleles are shown along the *x* and *y* axis, genotype frequencies (%) are shown on the vertical axis

The frequencies of ID polymorphism of 5-HTT gene in the male population sample meet the Hardy–Weinberg equilibrium. We found that the occurrence of D/D deletion genotype carriers is significantly (ninefold) higher among the male subjects with antisocial behavior (35%), as compared with the male general population sample (3.9%) (Fig. 1). The frequency of D/D genotype among the adolescents was 16.2%.

The carriers of D/D genotype had a higher risk to fall into the group of convicts as compared with the male subjects of the general population sample, with the odds ratio of ~13 (χ^2^ = 71.2; df = 1; *р* < 0.0001; OR = 13.43; CI: 6.52–27.67), The homozygous carriers of I/I insertion genotype are, though, encountered less frequently among the convicts, as compared with the general population.

A clear prevalence of the deletion allele carriers is also observed among the MMA fighters, being as high as 20.8% of cases, as compared with 3.9% in the general population sample. The carriers of D/D genotype had higher chances to be found in the group of MMA fighters as compared with the male subjects of the general population sample, with an odds ratio of ~6.5 (χ^2^ = 25.02; df = 1; *р* < 0.0001; CI: 2.86–14.73). A comparison of D/D genotype frequencies in the MMA fighter cohort and the control group of adolescents (20.8% vs. 16.2%) does not yield statistically significant difference (*р* = 0.29).

Thus, the comparative analysis of frequency distribution of ID genotypes of HTT gene shows a significant difference between the group of convicts and the control group (*р* < 0.0001).

A further classification of convict subjects into those committed violent or nonviolent crimes does not yield any difference in the frequencies of ID polymorphism, similarly to their subdivision into the first-time and habitual offenders.

### VNTR polymorphisms of serotonin transporter gene

**Fig. 2:**
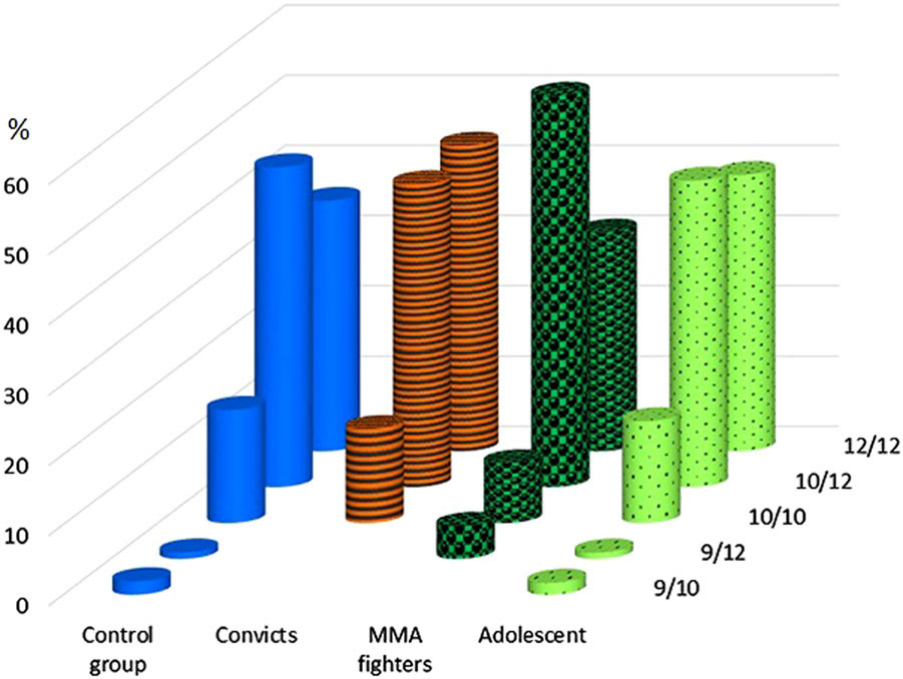
Genotype frequencies of 5-HTT VNTR polymorphism in the male subjects with antisocial behavior, the MMA fighters, the general population, and in adolescents. Study groups and combinations of VNTR alleles are shown along the *x* and *y* axis, genotype frequencies (%) are shown on the vertical axis

The 5-HTT VNTR polymorphism frequencies in the control sample meet the Hardy–Weinberg equilibrium. Among the convict subjects, the equal frequencies of 10/12 and 12/12 genotypes are observed (both are ~43%), while the genotypes 9/10 and 9/12 have never been encountered (Fig. 2). The increased frequency of 12/12 genotype carriers among the convicts is due to the contribution of those, who committed violent crimes (46% of cases, as compared with 35.6% in the general population). The carriers of 10/10 genotype were encountered slightly less frequently among the convicts, as compared with the general population.

The prevalence of 10/12 genotype was observed among the MMA fighters (55.7% vs. 45.5% in the control group), while the carriers of 10/10 genotype were encountered twice less frequently, than in the controls (8.5% vs. 16.1%). The carriers of 9/10 genotype were completely lacking among the MMA fighters, while 9/12 genotype was found in 4.7% of cases (*p* < 0.003).

In the adolescent group, the carriers of 10/12, 12/12, and 10/10 genotypes were occurring in 43.6%, 39.3%, and 14.5% of cases, respectively.

**Fig. 3:**
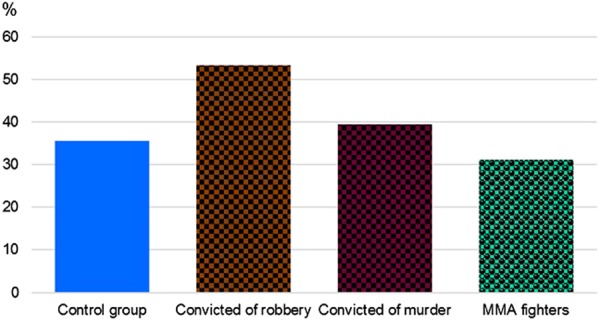
Frequencies of 12/12 VNTR 5-HTT genotype in the male subjects convicted of murder or robbery, in the general population, and in the MMA fighters. Study groups are shown along *x* axis, genotype frequencies of 12/12 VNTR 5-HTT (%) are shown on the vertical axis

In the subcohort of those convicted of extremely grave violent crimes, more than a half of subjects (53.3%) is represented by the carriers of 12/12 HTT genotype, while 40% of such carriers is found in the subcohort of those convicted of murder (Fig. 3).

**Fig. 4:**
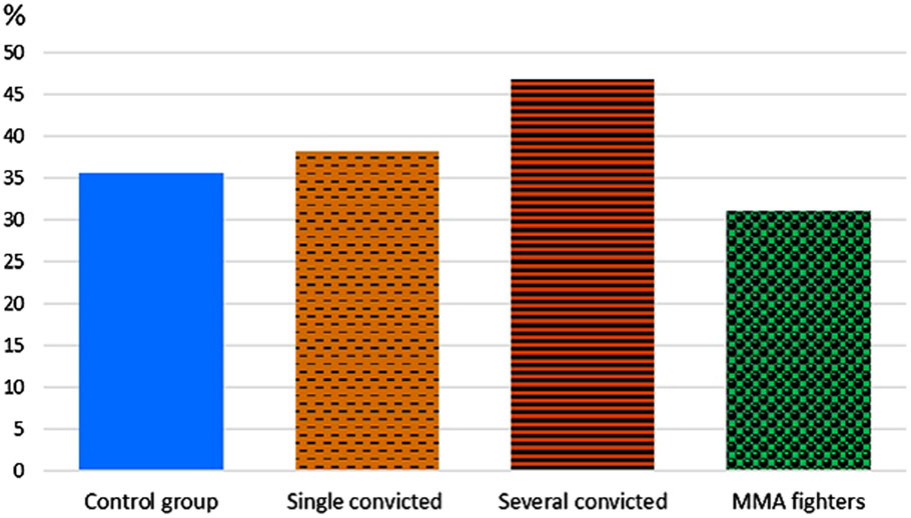
Frequencies of 12/12 VNTR 5-HTT genotype in the first-time and habitual offenders, the general population, and the MMA fighters. Study groups are shown along *x* axis, genotype frequencies of 12/12 VNTR 5-HTT (%) are shown on the vertical axis

When the cohort of convicts was further subdivided into the first-time and habitual offenders, a twofold higher frequency of homozygous 10/10 genotype carriers was observed among the latter (16.4% vs. 9.2%), while the carriers of 10/12 genotype were underrepresented in this subgroup (Fig. 4).

### Combination of ID and VNTR genotypes of 5-HTT gene

**Fig. 5:**
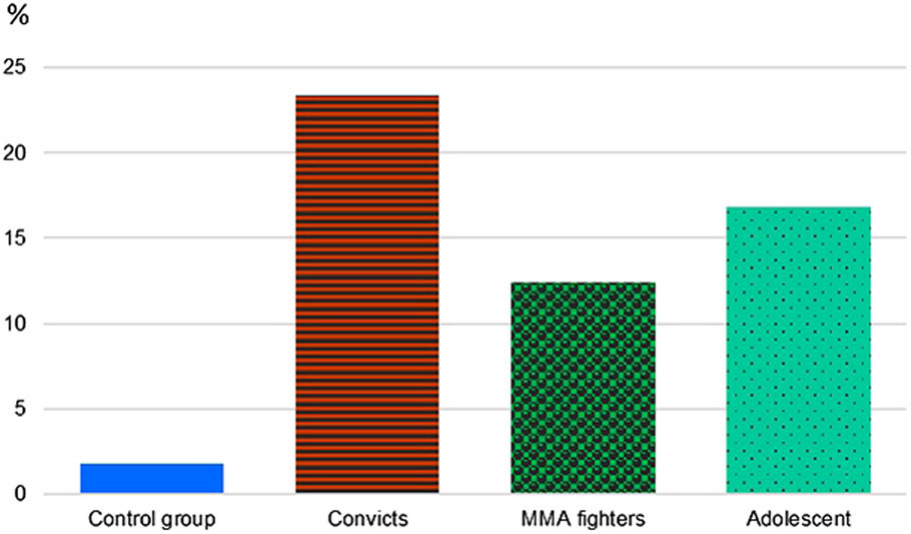
Combination of D/D and 12/12 5-HTT genotypes in the male subjects with antisocial behavior, the general population, the MMA fighters and in adolescents. Study groups are shown along *x* axis, genotype frequencies сombination of D/D and 12/12 5-HTT (%) are shown on the vertical axis

An analysis of the combination of ID and VNTR genotypes of 5-HTT gene (Fig. 5) demonstrates the increased frequency of the combination of D/D and 12/12 genotypes in the subjects with antisocial behavior (23.4% vs. 1.8% in the general population), along with the decreased frequency of the combination of I/I and 12/12 genotypes (4.3% vs. 18% in the general population).

The combination of I/D and 10/12 genotypes occurs more frequently among the convicts (26.3%), as compared with the general population (18.9%).

Thus, the male carriers of the combination of D/D and 12/12 5-HTT genotypes had higher risk to be found in the group of convicts, compared with the carriers of other genotypes, with an odds ratio of ~17 (χ^2^ = 37.8; df = 1; *p* < 0.001; OR = 17.1; CI: 6–48). Further subdivision of the convicts into those, who committed violent or nonviolent crimes, as well as the classification of subjects into those convicted of murder, or robbery, or the infliction of serious body injuries did not reveal the differences in the frequencies of particular combinations of 5-HTT ID and VNTR genotypes. Similarly, no such differences were observed between the subgroups of first-time and habitual offenders, as well as between the subgroups of subjects of different ethnic origin.

**Fig. 6:**
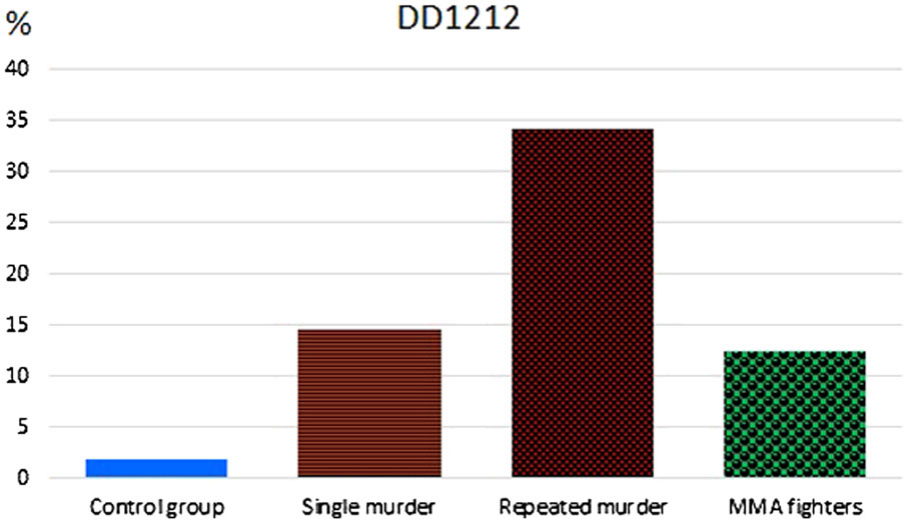
Combination of D/D and 12/12 5-HTT genotypes in subjects convicted of a single or repeated murder, the MMA fighters, and the general population. Study groups are shown along *x* axis, genotype frequencies сombination of D/D and 12/12 5-HTT (%) are shown on the vertical axis

However, when those convicted of single or repeated episodes of murder were analyzed separately, the combination of D/D and 12/12 genotypes was observed in 14.6% of the former, while in as much as 34.2% in the latter group (Fig. 6). The carrier of the combination of D/D and 12/12 genotypes had higher risk to be found in the subgroup of repeated murderers, than in the subgroup of those convicted of a single murder, with an odds ratio of ~17 (χ^2^ = 5.2; df = 1; *p* = 0.026; OR = 17.1; CI: 1.1–10.6).

The frequency of combination of D/D and 12/12 5-HTT genotypes among the MMA fighters is 12.4%, as compared with 1.8% in the general population. One should specify that this genotype combination is found in 12.9% of athletes practicing most hard combat style, while only in 11.4% of other fighters. The carrier of the combination of D/D and 12/12 genotypes had higher chance to be found among the MMA fighters, than in the male population sample, with an odds ratio of ~8 (χ^2^ = 16.8; df = 1; *р* = 0.001; OR = 7.9; CI: 2.5–24.9).

The frequency of this combination of genotypes in the group of adolescents is 8.8%.

Of note, the subjects with the combination of D/D and 12/12 genotypes may be underrepresented in the general population sample, since they may have refused to appear at the study, while being selected from the voters list, or may have been unable to do so due to their possible imprisonment at the moment of the study.

A psychological survey of the male convicts and the MMA fighters involved a personal interview with a qualified psychiatrist and testing with the use of different questionnaires. However, the two approaches yielded contrasting results. The fighters were providing frank answers in the interview, but they were presenting themselves exclusively positively when answering the test questions. It proved problematic to get the frank response to test questions from the convicts, since they were rather entertaining themselves by the participation in the survey. Even if they were providing frank answers, the results could hardly be considered objective, since in prison the male subjects develop stress reaction, which varies in expression depending on the term of imprisonment and a number of other factors. Further, a penitentiary-related personality deformation develops in the imprisoned male subjects due to the specifics of the penitentiary system (concentration of numbers of subjects with criminal behavior convicted of grave or extremely grave crimes)^26,27^.

## Discussion

Of note, the acts of violent behavior can differ in their origin, mechanisms, and their management. In research publications, the violent actions are often identified with the manifestation of aggression. Some authors consider aggression through the prism of dichotomy between impulsive and instrumental one—which means the variation from sharp response to a perceived threat or provocation to an intentional and targeted aggression^19,28,29^. One should not forget that aggressive behavior associated with a number of neurological and psychiatric disorders including neurodegenerative diseases is thought to be linked to the candidate genes involved in serotonin metabolism^6^. However, the study by Aluja et al.^30^ states that the aggressiveness does not play any role in the association between the polymorphisms of serotonin transporter gene and the impulsiveness-disinhibited personality.

The analysis of contribution of genetic and environmental factors to the development of persistent antisocial behavior established that the latter is mainly influenced by the genetic factors, the role of which is greatly increased with the age (above 15) both for the aggressive and the non-aggressive forms^31–33^. The study of the genetic and environmental stability and change in self-control in the children and young adults showed that the contribution of the genetic factors to the stability in self-control was ~74–92%, while they accounted for ~78–89% of the change in self-control, i.e, the “new” genetic variance^34^.

The research by Beaver et al.^35^, which investigates the factors, possibly, explaining why the volunteer-based military recruiting attracts some persons, while the others refuse to join, deserves special attention. The study revealed that 82% of the variance was the result of genetic factors, 18% of the variance was the result of non-shared environmental factors, and none of the variance was accounted for by shared environmental factors. The results presented suggest that the genetic factors are the major determinants of the development of antisocial behavior.

An analysis of publications on the association of serotonin transporter gene polymorphisms with behavioral features (apart from those involving a psychiatric pathology) reveals interesting opinions on this matter. For example, based on his research in the adolescents of Afro-American origin, Simons et al.^36^ suggests that the subjects bearing the deletion S-allele 5HTTLPR, as well as the carriers of L-allele DRD4 and L-allele MAOA, possess a high level of genetic plasticity. They express more violence in response to an unfavorable environment and, according to the scholar, less violence (compared with other genotypes) when the social environment is more favorable. This means that they are more sensitive to their social environment, than the other genotype carriers. For example, the subjects with the S-allele 5HTTLPR, as well, as the carriers of L-allele DRD4 and L-allele MOAO are more sensitive to their social environment, than the carriers of other genotypes. Thus, the authors postulate some degree of behavioral plasticity in response to the environment.

In our study cohorts, we noticed an important fact that the proportion of homozygous D/D deletion phenotype carriers in the male general population sample is reduced, as compared with the adolescents (3.9% vs. 16.2%). We also observed the accumulation of D/D genotype male carriers in the cohorts of subjects with extreme behavior—the group of MMA fighters and the comparison group of subjects with antisocial behavior. It appears that the carriers of D/D homozygous genotype are depleted in general population, while they accumulate in the groups of fighters and convicts.

The depletion of this genotype in the general population is, probably, due to their extreme behavior and, consequently, a higher risk to die in the conflict situations, traumas, accidents, etc., which are occurring more frequently in those subjects. Such persons, however, are being “conserved”, literally, due to the social microenvironment—in the penitentiaries, they are not only isolated from the society but also protected from the conflicts with the same subjects of criminal behavior (with the exception of such conflicts with the other inmates during imprisonment). The MMA fighters are immersed in the disciplined environment of their sports clubs from their childhood.

In our research, the behavior observed in the MMA fighters can be characterized as a harsh one. However, in a personal interview, the fighters demonstrate the level-headed and friendly behavior and deny their enhanced impulsiveness in appetence and behavior. Still, those fighters practicing the most brutal combat styles inform that they become irritable, engage in the heated discussions with their close relatives, and start feeling sleep problems when they have not been participating in fighting competitions for more than a month. While Liao and Hong^20^ suggest the link between the short S allele and the extreme violence, we propose that it can be associated with the violent behavior of noncriminal subjects capable of harsh responses without losing their self-control in particular situations. We believe that this deletion allele is influencing the serotonin metabolism, and it can be responsible for both brutal and “reasonably violent/harsh” behavior.

The published data presented in the Introduction link predisposition to aggression and violence, as well as the antisocial personality disorders, to allele 10 and genotype 10/10, however, we did not observe this association in our research. Similarly to our observations, a decreased frequency of 10-repeat VNTR 5-HTT allele was found among the Canadian children with extreme persistent and pervasive aggression^23^. Still, the chance to encounter the 10/10 genotype carrier among the repeated offenders is twofold higher, than among the first-time offenders.

As far as the HTT genotype combinations are concerned, the data obtained from the Spanish population indicate that the male penitentiary inmates with the combination of S/S + S/L and 12/12 5-НТТ genotypes have higher risk to fall into the group of subjects with antisocial personality disorder^37^. Also, this genotype combination is associated with «higher scores of the impulsive-disinhibited personality traits»^38^. Our data on the male subjects with antisocial behavior are in agreement with the results obtained in the Spanish population. A higher frequency of D/D and 12/12 genotype combination, as compared with the general population, is also found in the MMA fighters.

When studying the combinations of ID and VNTR genotypes of 5-HTT gene, we were attracted by a particular combination of serotonin transporter gene polymorphisms, which simultaneously harbors the deletion (D) allele of 5′-promotor region responsible for the low transcriptional activity of serotonin transporter gene and the 12-repeat VNTR allele located in intron 2 and coding for a highly functional serotonin transporter. It is the homozygous D/D + 12/12 genotype carriers that are overrepresented in the groups of males with extreme behavior, and it is exactly this combination that is found most frequently in the repeat murderers.

Since the 5-HTT allele with a promoter region deletion is associated with the decreased amount of functional active protein, the predisposition to extreme behavior, either deliberate or unconscious, may be due to this feature of serotonin neurotransmission.

In the fighters, we observe an association of harsh behavior, sometimes manifested in its most extreme form, with a controlled aggression during the combat. Phylogenetically, the manifestations of aggression were one of the survival factors and the options for obtaining and defending some privledges. According to Veroude et al.^39^, “During the early stages of human evolution, aggression was probably an adaptive trait, as it is for many animals in the wild today. It seems logical that during this period of time people who had the variants of genes that promoted aggression were more likely to survive than other people. These variants have persisted in the human genome and partly explain why some people exhibit aggressive behaviors”. Currently, an aggression manifested at particular moments is indispensable in the particular social groups, such as the military unit officers or the athletes practicing combat or power sports.

As far, as the phenotypes of male subjects under study are concerned, virtually, every fighter in our cohort represented an apparently hedonistic person, who was, however, complying with the socially acceptable norms, despite their high level of activity. Such fighters were capable of the situational pronounced aggressive reactions, for example, during the fight, which can be referred to as the “controlled aggression”. However, no aggression was detected in these fighters by the clinical psychiatric survey (Buss–Perry Aggression Questionnaire). Presumably, their predisposition to aggression is controllable either due to their education/upbringing or due to genetic factors.

The convicts were also driven by hedonist motifs in most cases, and their desires were often satisfied through illegal means. These male subjects often indicated at their ability to plan and organize an elaborate crime, however, they could be easily engaged into impulsive offences. All of them still recognized their actions as offenses.

As far as the self-control is concerned, Gottfredson and Hirschi^40^ propose the theory that “low self-control is the most important predictor for delinquent behavior”. According to our observations, the convicts belonging to the (inofficial) higher hierarchy casts in the prison possess sufficient self control (depending on circumstances).

Sun et al.^41^ makes a statement that *«*military size influences national violent-crime rates», as «it is found that countries with relatively larger size of military forces are more likely to have lower homicide rates». This may have a possible explanation that a proportion of subjects predisposed to extreme behavior and/or possessing the unstable social attitudes, but amenable to the disciplinary correction, may find themselves demanded/hirable in the military units. At the national level, this can be translated into a more successful fight against criminality.

The fighters turned out to share some of the studied gene polymorphisms with the males with antisocial behavior, while the other genetic traits contrasted the former to both general population and, which is more important, to the subjects predisposed to unlawful behavior. Thus, the fighters of our group and the convicts shared some genetic markers, presumably, responsible for the most efficient implementation of combat tasks, while generally their genotypes were different. Obviously, these similarities,as well as the differences will determine the route of personal development.

Thus, the study of allele and genotype frequencies of serotonin transporter gene polymorphisms in the male subjects with extreme behavior from the two compared cohorts of convicts and MMA fighters indicates that the carriers of homozygous D/D phenotype are accumulated and/or preserved in theses groups, while being eliminated from the general population. Their rare occurrence in the general population is due to their live habits associated with high health and life risks. The subjects convicted of grave violent crimes were the homozygous carriers of 12-repeat allele in about 50% of cases. The frequency of the combination of D/D and 12/12 genotypes in the studied groups is significantly higher than in general population. The occurrence of this combination is even higher in the groups of repeat offenders convicted of grave crimes or multiple murder. While a single murder committed by a subject may be due to the unfortunate circumstances, the repeated murder may reflect the established pattern of criminal behavior. Predisposition of MMA fighters to violent behavior and physical aggressive suppression of the opponent is successfully realized in their professional career, as it has a positive effect on their combat efficiency. However, this pattern of behavior is controlled in the MMA fighters. While studying the male fighters, we observed an assortative grouping in their teams, since they tended to form the groups of subjects with most similar genotypes, both those under study here or in the other research projects of our group. The formation of such groups among the convicts was technically much less traceable. We suggest that the genetic features of the genes involved in the functioning and compensatory potential of neurotransmitter system are manifested phenotypically as the particular types of temperament, patterns of behavior or mindsets. In general, these psychological and behavioral features can be translated into social realization, either normal or pathological. It needs to be specified that we were aiming at minimizing the influence of social environment by enrolling the study group participants from those, who were raised in a favorable environment in childhood. Thus, the study of genetic behavioral features is rather interesting and promising both scientifically and for the establishment of proper state policies, since in the future it can help to determine the factors favoring either criminalization or the development of fighting capacities without a predisposition to antisocial behavior.
